# Assessing stress among medical students in Anbar governorate, Iraq: a cross-sectional study

**DOI:** 10.11604/pamj.2018.31.96.16737

**Published:** 2018-10-09

**Authors:** Ameel Farooq Al Shawi, Abdulrahman Nassir Abdullateef, Mohammad Anmar Khedher, Mohammad Saadon Rejab, Russul Nadhim Khaleel

**Affiliations:** 1 Department of Community Medicine, College of Medicine, University of Falluja, Iraq

**Keywords:** Stress, medical students, anbar governorate, Falluja University, Iraq

## Abstract

**Introduction:**

A high prevalence of stress has been recorded among medical students worldwide. Additionally, high levels of personal distress may have a negative effect on the cognitive functioning and learning abilities of medical students.

**Methods:**

A cross-sectional study was conducted using medical students in the Al-Anbar governorate; data collection was carried out from February to March 2018. The assessment of stress levels among these students was administered using the Kessler10 Psychological Distress instrument (K10).

**Results:**

231 students (77.5%) reported at least some degree of stress, ranging between severe (30.2%), moderate (25.5%) and mild (21.8%). The highest proportion of students with stress (11.5%) was reported by first-year students; this proportion largely fell within the moderate and severe categories. There was a significant statistical association between gender and stress level.

**Conclusion:**

There is a high level of stress among medical students in Falluja and Anbar Universities; this may require special programmes to identify any predisposing factors.

## Introduction

Stress can be defined as an uncomfortable emotional experience that is accompanied by predictable biochemical, physiological and behavioural changes [1]. The psychological and physical state occurs when the capabilities of the individual are not sufficient to cope with the requirements of their current situation [2]. This may lead to feelings of fear, incompetence, anger, aggression and guilt and, if unresolved, may even lead to associated physical and psychological morbidities. Individuals may, however, respond differently to the same situation [3, 4]. Stress is a normal and beneficial part of life that helps individuals to learn. An optimal level of stress enhances learning, whereas excess levels of stress can cause health problems, such a condition may lead to reductions in students' level of self-esteem and can negatively affect their academic achievement. A high level of stress may have a negative effect on the cognitive functioning and learning abilities of medical students. Moreover, stress and depression have consistently been linked to detrimental mental and physical health effects [5, 6]. Due to the competitive nature of medical school, medical students may perceive higher levels of stress than non-medical students [7]. Medical students, especially freshmen, are a group particularly prone to stressors related to the transitional nature of college life [8, 9]. They must adjust to being away from home for the first time, maintain a high level of academic achievement and adjust to new social environments. Stressors do not cause anxiety or tension by themselves; instead, stress results from stressors found in these environments and the individual's perception and reaction to those stressors [10]. The amount of stress may be influenced by the individual's resources, which subsequently determine their coping mechanisms for stressful events and situations [8]. In medical colleges, the first year is the beginning of students' medical journey; with it comes a disruptive of environment with the realisation that it is impossible to master the learning process professionally [11]. Period of the university life is critical period during that the students are more vulnerable for psychological disorders such as depression and anxiety [12, 13]. A prevalence of stress has been recorded among medical students worldwide, with levels of stress in medical students ranging high from 25-90% [3, 14-16]. Additionally, high levels of stress were observed among junior doctors and these are believed to have originated in their undergraduate studies [17]. More psychological stress has been reported among female medical students than in their male counterparts, as women are more vulnerable to psychological distress [18-20]. The objectives of this study: to assess levels of stress among medical students in Anbar governorate, Iraq, and to assess the association of perceived stress with students' sociodemographic characteristics.

## Methods

**Study design:** A cross-sectional study was conducted among medical students in the medical colleges of two universities in the Al-Anbar governorate in the west of Iraq: Fallujah and Anbar.

**Instruments:** The questionnaire was developed by a community physician; it consisted of questions covering sociodemographic characteristics, including age, gender, year of study, household monthly income, father's education and the Kessler10 Psychological Distress instrument (K10) developed by Kessler and colleagues [21] to address psychological distress. The K10 instrument has been widely used in population-based epidemiological studies to measure current (1-month) distress and to measure participants' level of stress associated with psychological symptoms in population surveys. It was used in the World Health Organization's World Mental Health Survey as a clinical outcome measure, and had good validity and reliability [22, 23]. The K10 consists of 10 questions in the form of, “How often in the past month did you feel …”, and offers specific symptoms, such as, “tired for no good reason”, “nervous”, “sad” or, “depressed”. The five possible responses for each question range from, “None of the time” to, “All of the time”, and were scored from 1 to 5, respectively. All of the questions were collated to obtain a total score. The total score was interpreted as follows: a score of less than 20 was considered to represent the absence of stress, a score of 20-24 represented mild stress, 25-29 represented moderate stress and 30-50 represented severe stress [24].

**Data collection:** This was carried out from February to March 2018, and took the form of a closed, self-administered survey.

**Sampling technique:** The sample was collected by dividing each college into 5 study levels; participants were involved conveniently from each level.

**Study subjects:** The questionnaire form was distributed to 500 male and female students from the medical colleges of both universities.

**Data analysis:** Data entry and statistical analysis was conducted using the Excel program and SPSS Version 20; chi square test was used to measure the association between level of stress and other variables such as sociodemographic characteristics. P < 0.05 was considered to be a significant result.

**Ethical consideration:** Ethical approval for this study was obtained from the scientific committee of the College of Medicine, University of Falluja. Participants were given the choice to participate in the study and were informed that all the information taken would be kept strictly confidential and would only be used for research purposes. Verbal consent was obtained from the participants, who were permitted to respond in their own time and privacy, after researchers explained the aim of the research.

## Results

Of the initial 500 participants, only 300 responded, giving a response rate of 60%. The age of participants was between 17 and 27 years (mean, SD 20+-2.2); 117 (39%) were male; 286 (95%) were single; and 5 (1.7%) were married. Regarding the monthly household income, 19.3% had an income between 1000.000-1500.000 Iraqi Dinars (IQD) and just 6.7% fell into the >2000.000 IQD category (Table 1). The results showed that 231 of the students (77.5%) were stressed; severe stress was reported by 90 (30.2%) of the subjects; moderate stress was observed in 76 (25.5%); and mild stress was reported by 65 (21.8%) participants (Table 2). 53.4% of the stressed students were female and 24.14% were male; there was a significant statistical association between gender and stress level. Severe stress was mentioned by 64 (21.7%) females and 25 (8.5%) males; moderate stress was reported by 49 (16.6%) females and 25 (8.5%) males; and mild stress was reported by 44 (14.9%) females and 21 (7.14%) males (see Table 3). The highest proportion (11.5%) of stressed students were in their first year of study, most of whom reported moderate and severe stress; on the other hand, the lowest proportion (0.37%) of stress was reported by a sixth-year student, and fell into the mild stress category, as shown in Table 3. 11.27% of students fell into the 500.000-1000.000 Iraqi Dinars (IQD) (420-850$) income category, reporting severe stress, while just 1.5% fell into the 1500.000-2000.000 IQD (1250-1660$) income category, reporting mild stress. The highest and lowest proportions in each type of income were: <500.000 IQD (420$), most of whom (6.67%) were in the moderate stress category, whereas the fewest (3.75%) fell into the mild stress and no stress categories. In the 500.000-1000.000 IQD (420-850$) income category, 11.27% fell into the severe stress category; just 8.64% were in the moderate stress category. In the 1000.000-1500.000 IQD (850$-1250$) income category, 6.76% were in the no stress category and only 3.75% were in the severe stress category. In the 1500.000-2000.0000 IQD income category, 4.5% were in the severe stress category and 1.5% were in the mild stress category. In the 2000.000 IQD (1660$) income category, 3% reported severe stress and 1.5% reported either no stress, or mild or moderate stress. Over, 28.9% experienced severe stress, and just 22.55% experienced mild stress. However, there was a statistically insignificant association between monthly income and level of stress; similar insignificant statistical associations were found between parents' education and students' level of stress; more details are shown in Table 3.

**Table 1:** Sociodemographic characteristics of the participants

	Type	Frequency
Gender	Male	117
	Female	179
	Total	296*
Level of study	1st stage	114
	2nd stage	28
	3rd stage	49
	4th stage	38
	5th stage	28
	6th stage	12
	Total	269*
Marital status	Single	286
	Married	5
	Total	291*
Monthly Household Income	< 500.000 IQD	55
	1000.000-1500.000 IQD	58
	1500.000-2000.000 IQD	28
	> 2000.000 IQD	20
	Total	267*

* The total <300 due to missed answers

**Table 2:** Students’ stress scores

Level of stress	**Type**	**Frequency**	**Percentage**
	No stress	67	22.5%
	Mild stress	65	21.8%
	Moderate stress	76	25.5%
	Severe stress	90	30.2%
	Total	298*	100%

* The total <300 due to missed answers.

**Table 3:** Association between sociodemographic characteristics and stress scores

		Score level				Total	P** value
		No stress	Mild	Moderate	Severe		
Gender	Male	45	21	25	25	116	0.00
	Female	21	44	49	64	178	
Total		66	65	74	89	294	
Stage of college (Level of study)	1st stage	25	26	31	31	113	
	2nd stage	5	5	8	10	28	0.57
	3rd stage	8	10	18	13	49	
	4th stage	10	14	6	8	38	
	5th stage	6	5	8	9	28	
	6th stage	4	1	2	5	12	
Total		58	61	73	76	268*	
Father’s occupation	Employee	41	38	41	57	177	0.5
	Worker	14	15	14	14	57	
	Retired	11	10	15	16	52	
	Unemployed	0	1	3	0	4	
Total		66	64	73	87	290*	
Father’s education	Higher	11	10	8	20	49	O.8
	College	42	39	50	51	182	
	Secondary	13	12	11	14	50	
	Primary	1	2	3	3	9	
Total		67	63	72	88	290*	
Mother’s education	Higher	4	5	4	7	20	0.6
	College	23	22	30	40	115	
	Secondary	26	18	20	25	89	
	Primary	12	18	17	15	62	
Total		65	63	71	87	286*	
Income***	< 500.000 IQD.	10	10	18	17	55	0.4
	500.000-1000.000 IQD.	25	27	23	30	105	
	1000.000-1500.000 IQD.	18	15	15	10	58	
	1500.000-2000.000 IQD.	7	4	5	12	28	
	> 2000.000 IQD.	4	4	4	8	20	
Total		64	60	65	77	266*	

* The total <300 due to missed answers.

** P value for chi square test

*** 1 USD equal to 120 IQD

## Discussion

There was a high prevalence of stress among medical students in the current study; this finding was higher than the results of studies in Saudi Arabia (57%) [25], in the Islamic Republic of Iran (61.3%) [3] and in the United Kingdom (31.2%) [26]. A significant statistical association between gender and stress level as female students were more stressed than male students, this finding was consistent with what was reported in other studies that showing women were more vulnerable for psychological distress [20]. The prevalence of stress, ranked by students' level of study, was highest in the first year, followed by the third year and subsequently the sixth year. However, this result may be skewed by first-year students facing a new academic environment and being inundated with information. This finding was consistent with what was reported in other studies, demonstrating that stress was more prevalent in first-year students and declined as students progressed through medical school, with the exception of their final year, in which students reported a high level of stress [27, 28]. Medical students have a limited time to understand and memorise the information they study, and the huge amount of information combined with the state of recurrent exams may create feelings of distress and disappointment, as students may not pass every exam in their curriculum [29]. This finding may also be attributed to the course workload, lack of leisure time, defective learning materials and frequent examinations. Progressive assessments in anatomy physiology and biochemistry increase students' stress levels, as they are required to pass these courses to progress to the next level of study [30, 31]. Often, mounting academic pressure and the limited time they are given to acquire this vast knowledge prevents medical students from adopting a healthy lifestyle. Though there was statistically insignificant association between level of the study and stress severity, additionally there were statistically insignificant associations between parent's education, family monthly income with stress severity. These findings might be due to the unstable environment of Iraq as the general population exposed to different kinds of violence and traumatic events especially during last two decades. The excessive amount of stress in medical training predisposes students to experience difficulties when solving problems, reduces concentration and, finally, increases their chances of developing depression [32]. It is important to mention that high stress levels can reduce students' mental stability, impair their judgment and increase absenteeism. In effect, all these things compromise the academic achievement of students and prevent them from succeeding and improving their skillset. Excessive stress results in excessive secretions of cortisol, the stress hormone, causing a decrement in the memory retrieval functions of the hippocampus and amygdala due to the blockage of glucocorticoid receptors, thus facilitating the aforementioned symptoms [32]. The prevalence of stress according to monthly household income was found in students with incomes of < 500.000 IQD; this might be attributed to the fact that students are facing financial problems in addition to their academic ones, as they need money for basic things like clothes, food and photocopying.

## Conclusion

There is a high level of stress among medical students in Falluja and Anbar Universities, which may require special programmes to address the identified predisposing factors.

**Recommendation:** Creating a positive teaching environment in medical colleges to reduce students' levels of stress.

### What is known about this topic

- Medical Students expose to stress during their studies.

### What this study adds

- High level of stress among medical students in Anbar and Falluja universities, Iraq;
- Stress levels were higher in females compared to males;
- There was a difference of stress level according to the level of the study.

## Competing interests

The author declare no competing interest.
